# Supplementary material for: Manipulating microRNA miR408 enhances both biomass yield and saccharification efficiency in poplar
Source: Nat Commun. 2023 Jul 18;14:4285. doi: 10.1038/s41467-023-39930-3 (PMC10354043; doi:10.1038/s41467-023-39930-3)
Supplement: Supplementary file 1 — Supplementary Information [file 41467_2023_39930_MOESM1_ESM.pdf]

Manipulating microRNA *miR408* enhances both biomass yield and saccharification efficiency in poplar

Guo *et al.*

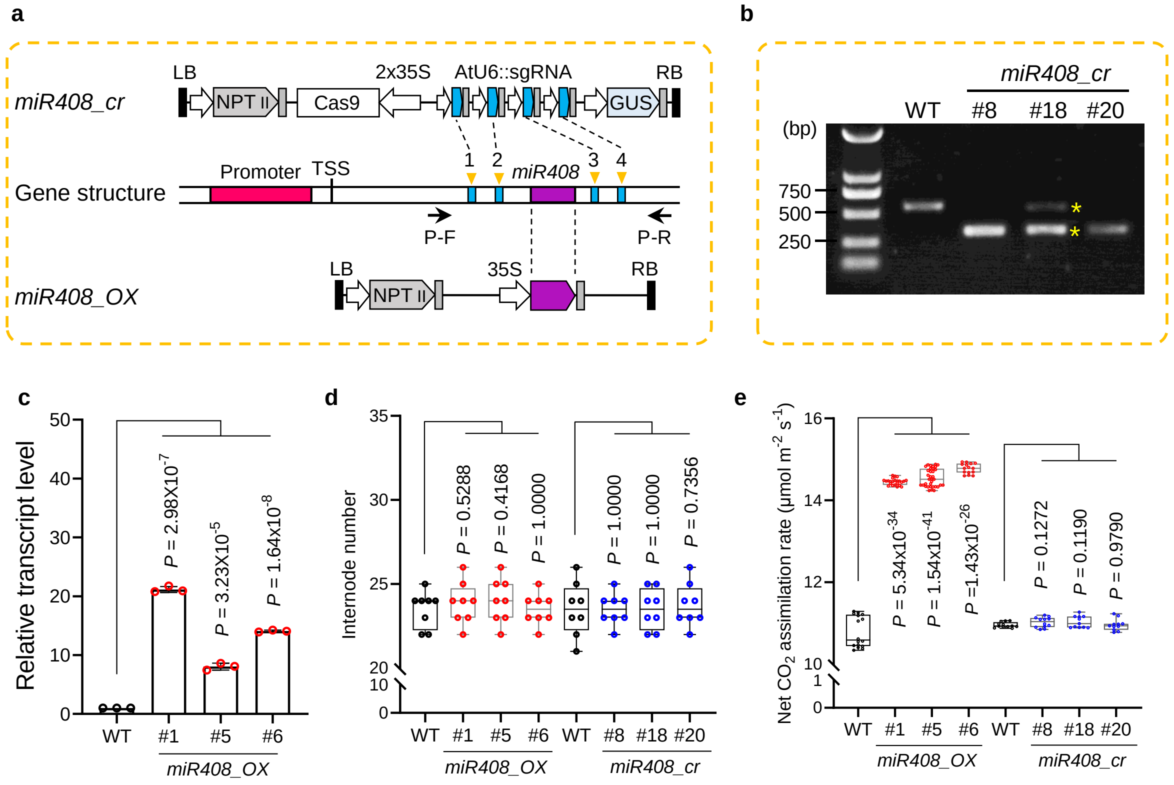

Supplementary Figure 1. *miR408* vector construction and identification of transgenic plants.

(**a**) Schematic diagram showing the genomic structures of *miR408* and the construction of *miR408_cr* and *miR408_OX* vectors. The four orange triangles (two upstream and two downstream of the *miR408* sequence) indicate the four conserved gRNA target sites designed to knock out *miR408*. P-F and P-R are the primers used for PCR identification of *miR408_cr* lines.

(**b**) Identification of *miR408_cr* lines using P-F and P-R primers. The length of the amplified fragment in WT was 527 bp, whereas that in #8 and #20 lacked approximately 218 bp. There were two bands in #18, the lower one (marked in yellow star) is similar to #8 and #20, and the upper one is similar to WT. Three samples each were analyzed with similar results.

(**c**) qRT-PCR analysis of mature *miR408* in stems. Values are means ± SD (n = 3, two-tailed Student's *t*-tests, n represents 3 trees sampled respectively from each transgenic line).

(**d-e**) Comparisons of internode number (**d**) and net CO_2 assimilation rate (**e**) in WT, overexpression and knock-out lines. The upper and lower whisker represents the maximum and minimum value, respectively. The upper, lower and middle box lines represent the two quartiles and median of values in each group. All *P*-values are from two-sided Student's *t*-tests. The numbers represent trees sampled respectively from each transgenic line are as follows: d = 8, e =11 (#18 and #20), 12 (#8), 16 (WT for *miR408_OX*), 12 (WT for *miR408_cr*), 23 (#1), 35(#5), 15 (#6).

Source data are provided as a Source Data file.

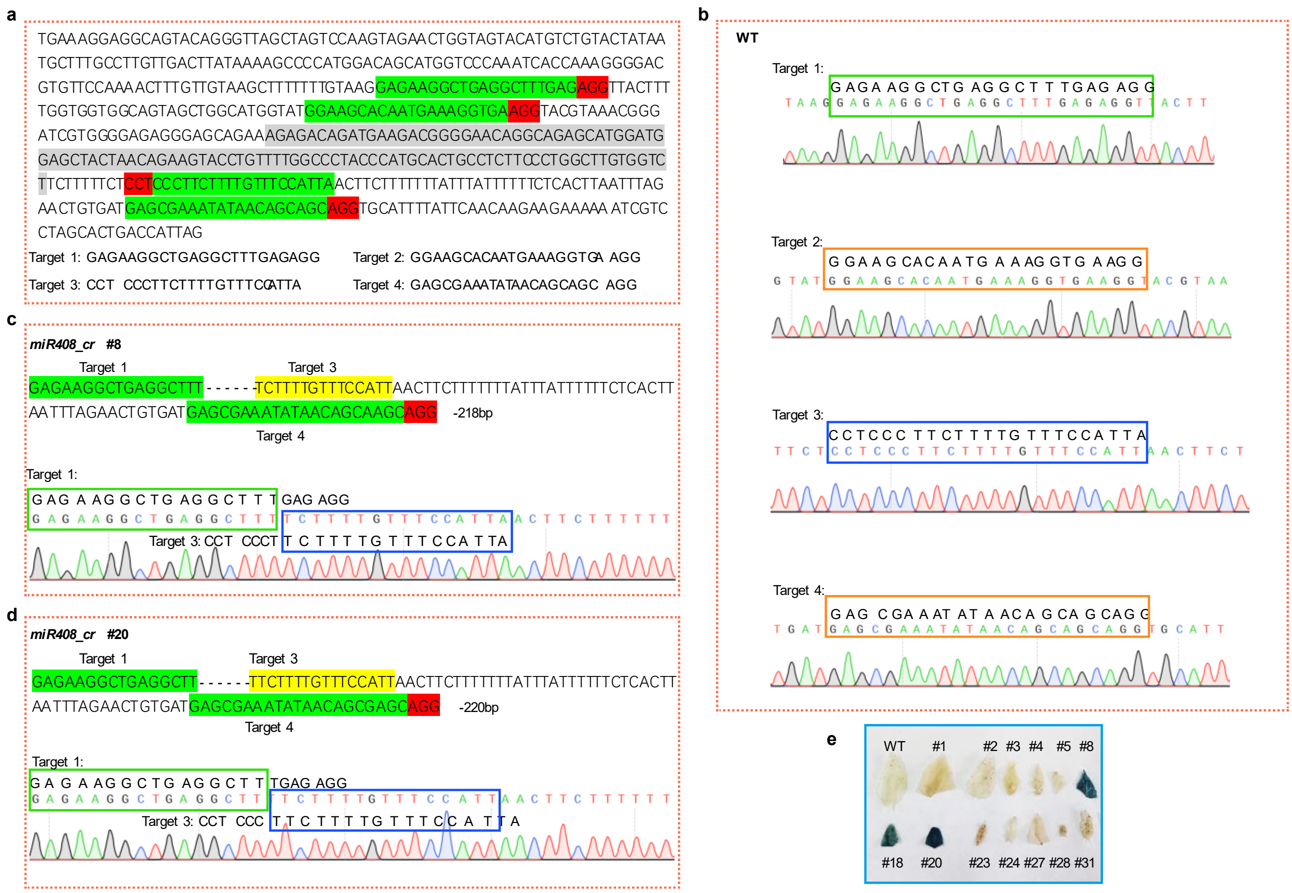

Supplementary Figure 2. Identification of *miR408_cr* poplar.

(**a**) The genome sequence of *miR408* (Grey shading) and its upstream and downstream sequences. Green and red shading indicated the four targets.

(**b**) The diagram of the four targets in WT.

(**c-d**) Sequences of PCR amplified upstream and downstream sequences of *miR408* precursor in *miR408_cr* #8 and #20, indicating confirmed gene editing.

(**e**) GUS staining of regenerated *miR408* knockout poplar. The negative control was WT leaves, and GUS staining was positive in #8, #18, #20, indicating that they were transgenic plants, whereas GUS staining was negative in #1, 2, 3, 4, 5, 23, 34, 27, 28, 31, suggesting that they were non-transgenic poplars.

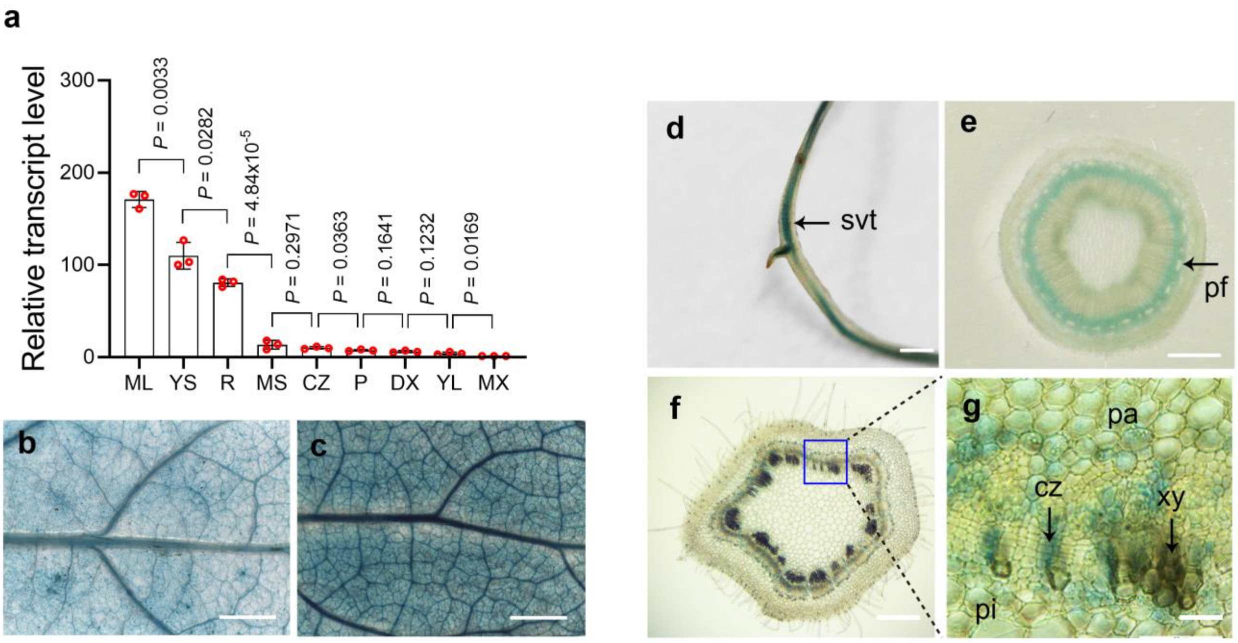

Supplementary Figure 3. Expression pattern of *Pag-miR408*.

(**a**) qRT-PCR analysis of the expression of mature *miR408* in different tissues. 5S rRNA was used as the endogenous control to normalize the relative transcript levels of *miR408*. Values are means ± SD (n = 3, two-tailed Student's t-tests, n represents 3 trees sampled of WT). R, root; YS, young stem; MS, mature stem; YL, young leaves; ML, mature leaves; P, phloem; DX, developing xylem; CZ, cambium zone; MX, mature xylem.

(**b-c**) *pmiR408::GUS* expression pattern in poplar. The promoter region of *miR408* was fused to the GUS reporter gene to generate *pmiR408::GUS* transgenic plants in the WT background. Tissues at different developmental stages were stained for GUS activity: b, young leaf; c, mature leaf; Scale bar, 1 mm (**b**, **c**).

(**d-g**) Tissues at different developmental stages were stained for GUS activity: root (**d**, bar, 5 mm); cross-section of mature stem (**e**, bar, 1 mm); cross-section of young stem (**f**, bar, 500 μm); enlarged young stem (**g**, bar, 25 μm). Three samples each were analyzed with similar results for **b**-**f**.

Source data are provided as a Source Data file.

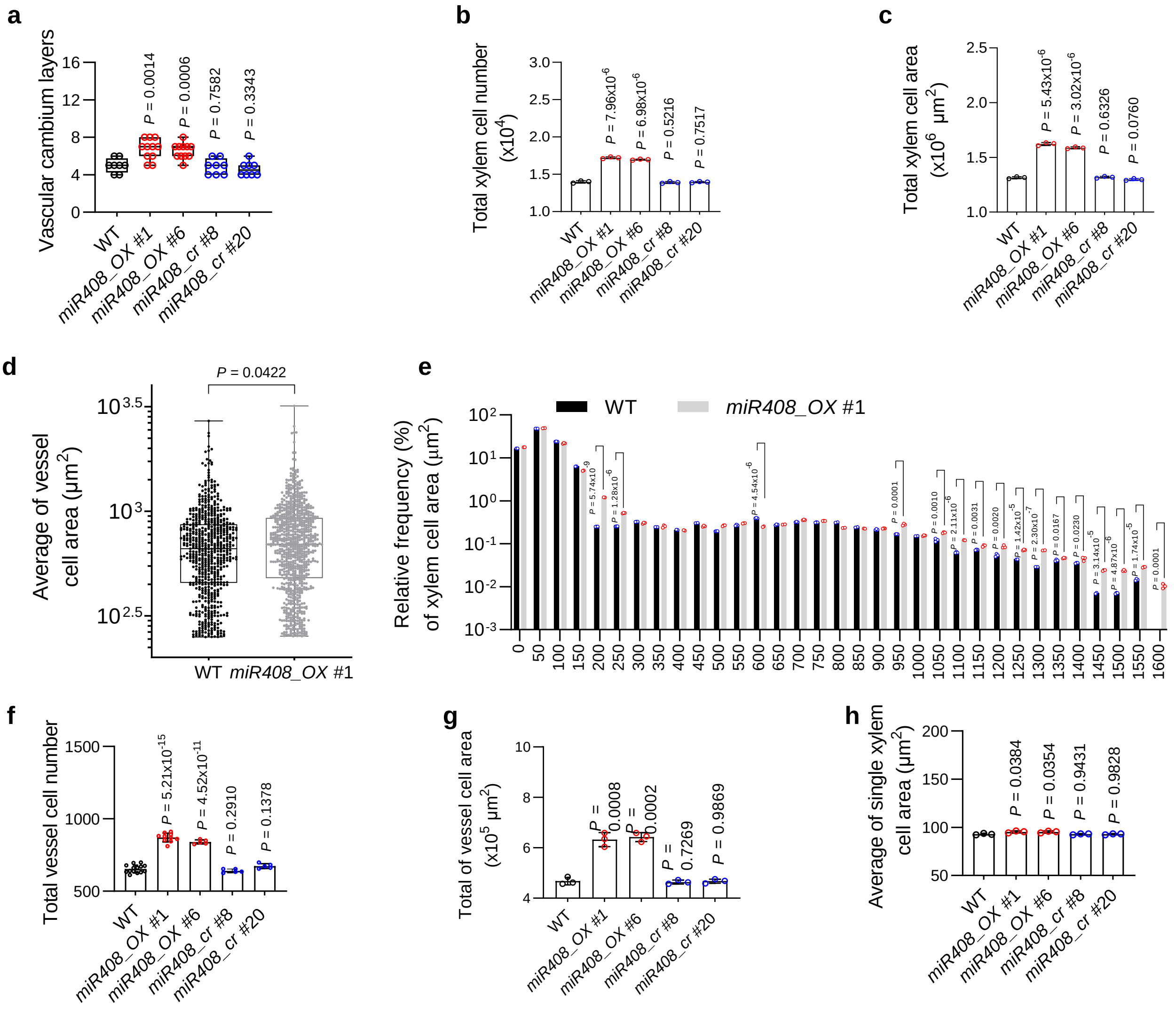

Supplementary Figure 4. Anatomical analysis of stems of *miR408* overexpression and knockout lines.

(**a-d**) Statistical analysis of vascular cambium layers, total xylem cell number, total xylem cell area and average of vessel cell area.

(**e**) Relative frequency of xylem cell area. 0-200 μm^2 represents fiber cells; 250-1600 μm^2 represents vessel cells.

(**f-h**) Statistical analysis of average of total vessel cell number, total vessel cell area and average of single xylem cell area. The numbers of individual trees sampled respectively from WT and each transgenic lines are as follows: **a**=8-11; **b-e**, **g-h**, n = 3; f, n=5-14. All *P*-values are from two-tailed Student's *t*-tests. Values are means ± SD (**b-c**, **e-h**). The upper and lower whisker represents the maximum and minimum value, respectively. The upper, lower and middle box lines represent the two quartiles and median of values in each group (**a**, **d**). Source data are provided as a Source Data file.

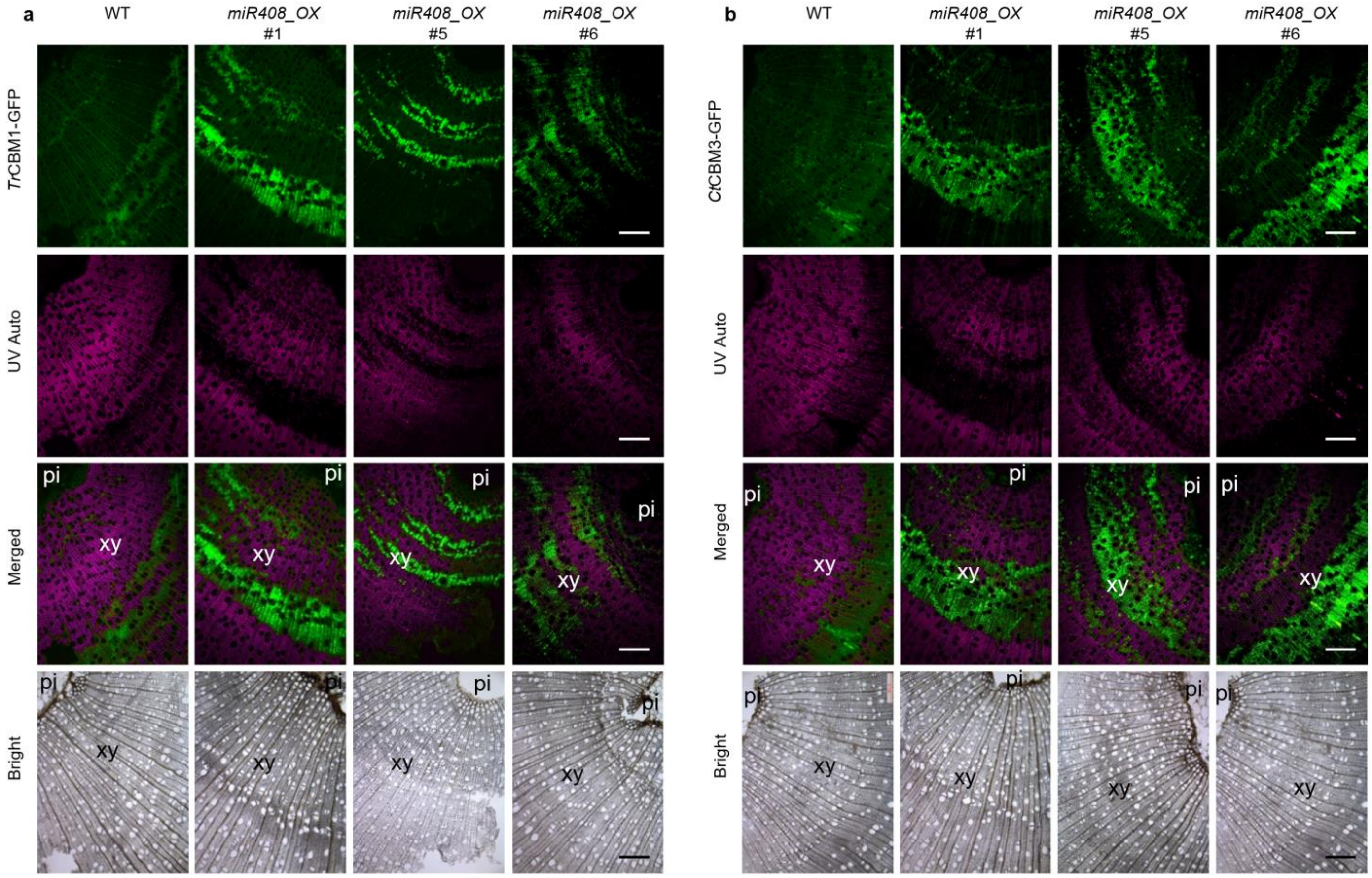

Supplementary Figure 5. Fluorescence microscopy of cell walls exposed to *Tr*CBM1-GFP (a) and *Ct*CBM3-GFP (b). Transverse sections of basal stems of one-year-old naturally dried poplars. *Ct*CBM3 and *Tr*CBM1 specifically recognizes cellulose and the probe exhibits green fluorescence. Autofluorescence (red) under UV shows lignin and the merged images highlight the negative correlation between probe binding and autofluorescence. Pi, pith; xy, xylem. Scale bars, 200 μm. Three samples each were analyzed with similar results for **a**, **b**.

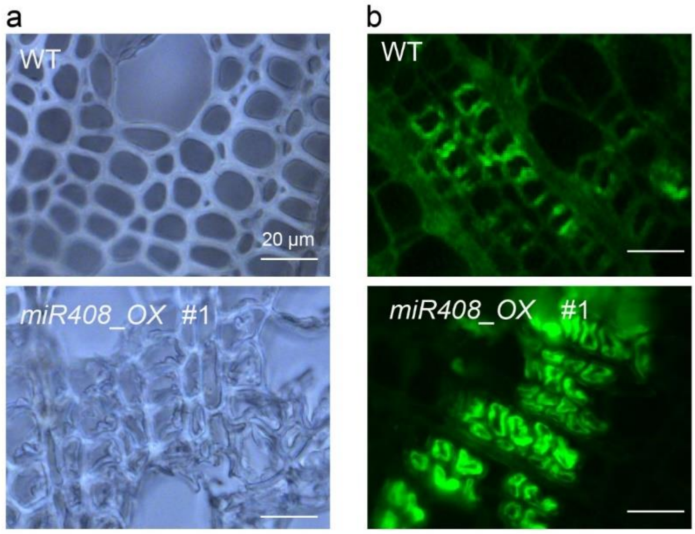

Supplementary Figure 6. Labeling of stem cross sections of one-year-old naturally dried poplars with a microbial cellulose-binding module.

(**a**) Bright field images of WT and *miR408* overexpression poplars.

(**b**) Fluorescence microscopy of cell walls exposed to *Ct*CBM3-GFP. The naturally dried cells of one-year-old stem of *miR408_OX* #1 that were strongly labeled with green fluorescence were also collapsed due to water loss. Scale bars, 20 µm. Three samples each were analyzed with similar results for **a**, **b**.

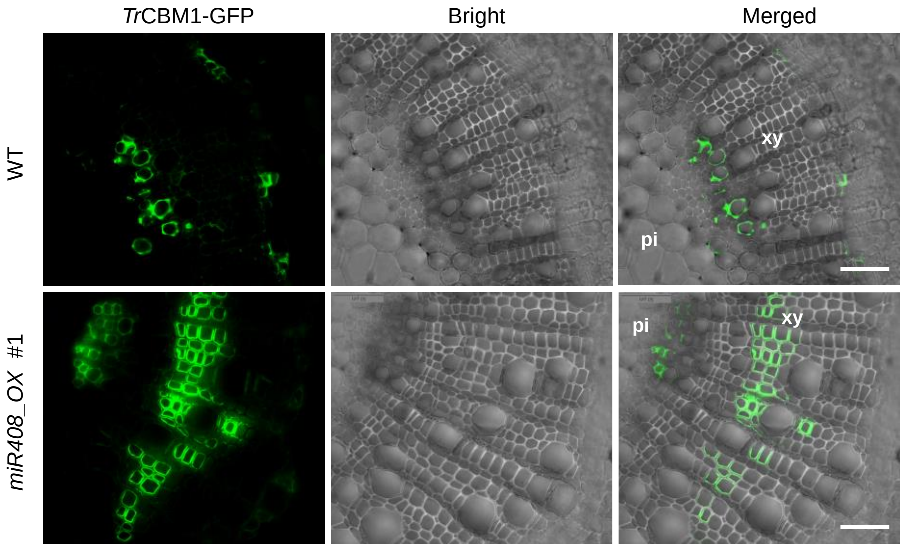

Supplementary Figure 7. Fluorescence microscopy of cell walls exposed to *Ct*CBM1-GFP in transverse sections of basal stems of two-month-old tissue-cultured poplars. The *Ct*CBM1 signal was present in the xylem near the pith in WT whereas it covered much more of the xylem area in *miR408_OX* #1. The xylem vessels and fiber cells of *miR408_OX* #1 plants were also enlarged. Scale bars, 50 μm. Three samples each were analyzed with similar results.

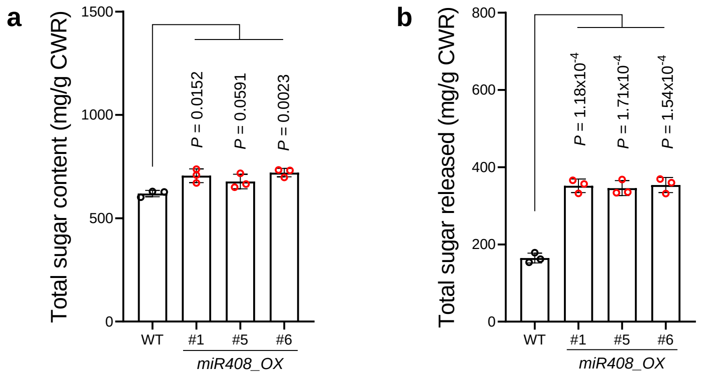

Supplementary Figure 8. Overexpression of *miR408* strongly enhances saccharification efficiency.

(a) Total sugar content of cell wall residue.

(**b**) Total sugar released. Values are presented as means ± SD (n = 3, *P*-value is from two-tailed Student's *t*-test, n represents 3 trees sampled respectively from each transgenic line). Source data are provided as a Source Data file.

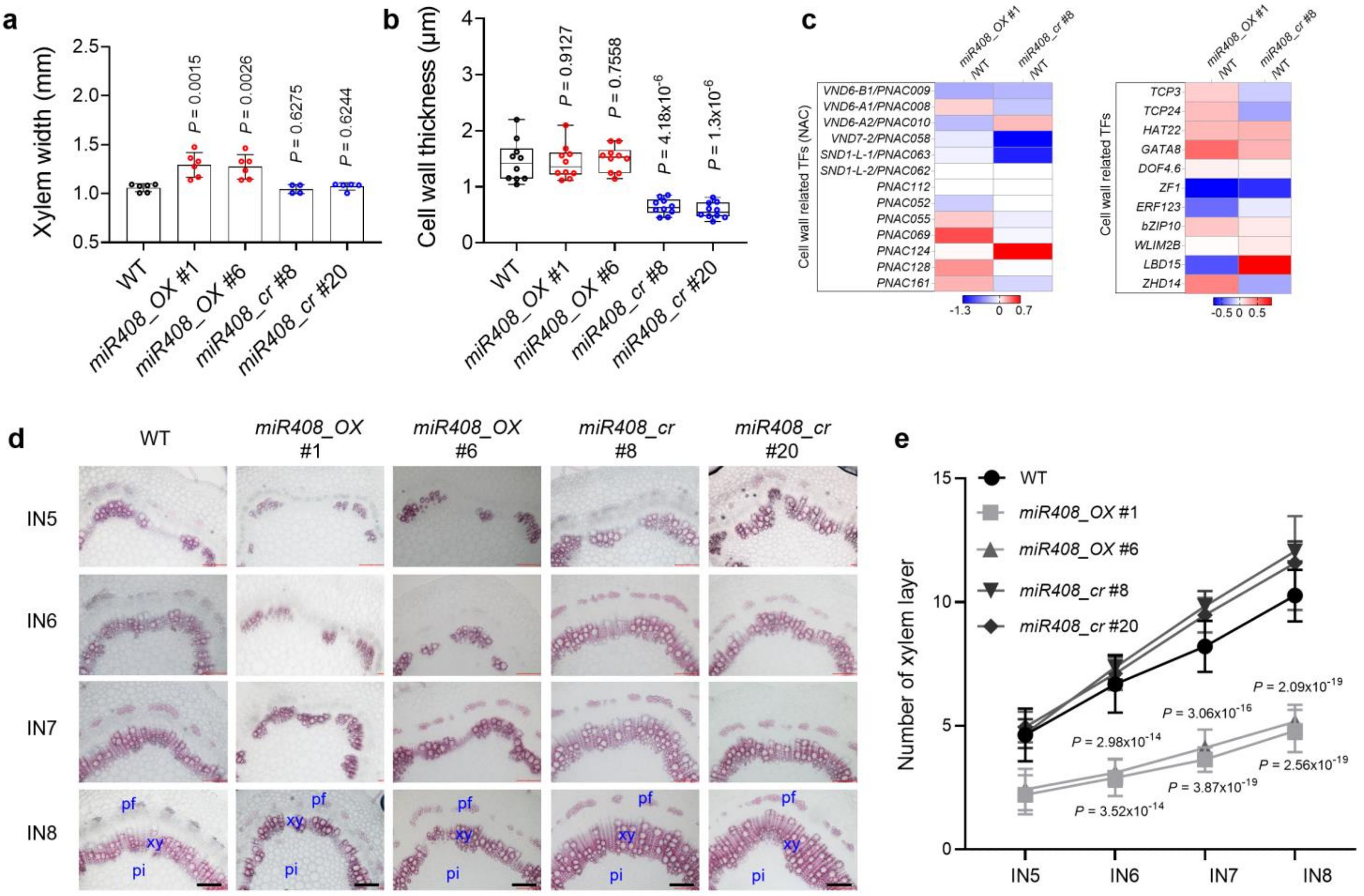

Supplementary Figure 9. Cross-sections of *miR408_OX* and knockout poplar visualized by phloroglucinol staining.

(**a**) Statistical analysis showing xylem width of WT, overexpression and knockout lines.

(**b**) Statistical analysis showing secondary cell wall thickness of vessels and fibers in xylem of *miR408* overexpression, knockout and WT plants. The upper and lower whisker represents the maximum and minimum value, respectively. The upper, lower and middle box lines represent the two quartiles and median of values in each group.

(**c**) Heatmap illustrating the transcript levels of cell wall related TFs.

(**d**) Cross-sections from IN5, IN6, IN7, IN8. Phloem starts to appear at IN5 of WT and knockout poplars but not until IN7 of *miR408_OX* plants. ph, phloem; xy, xylem; pi, pith. Scale bars, 100 μm.

(**e**) Estimation of number of lignified xylem cell layers using phloroglucinol staining. Statistical analysis showed reduced numbers of lignified xylem cell layers in *miR408_OX* compared with WT, while increased numbers in knockout poplars. The numbers of individual trees sampled respectively from WT and each transgenic lines are as follows: a=4-6; b, n = 10; d, n=19. All *P*-values are from two-tailed Student's *t*-tests. Values are means ± SD (**a**, **e**).

Source data are provided as a Source Data file.

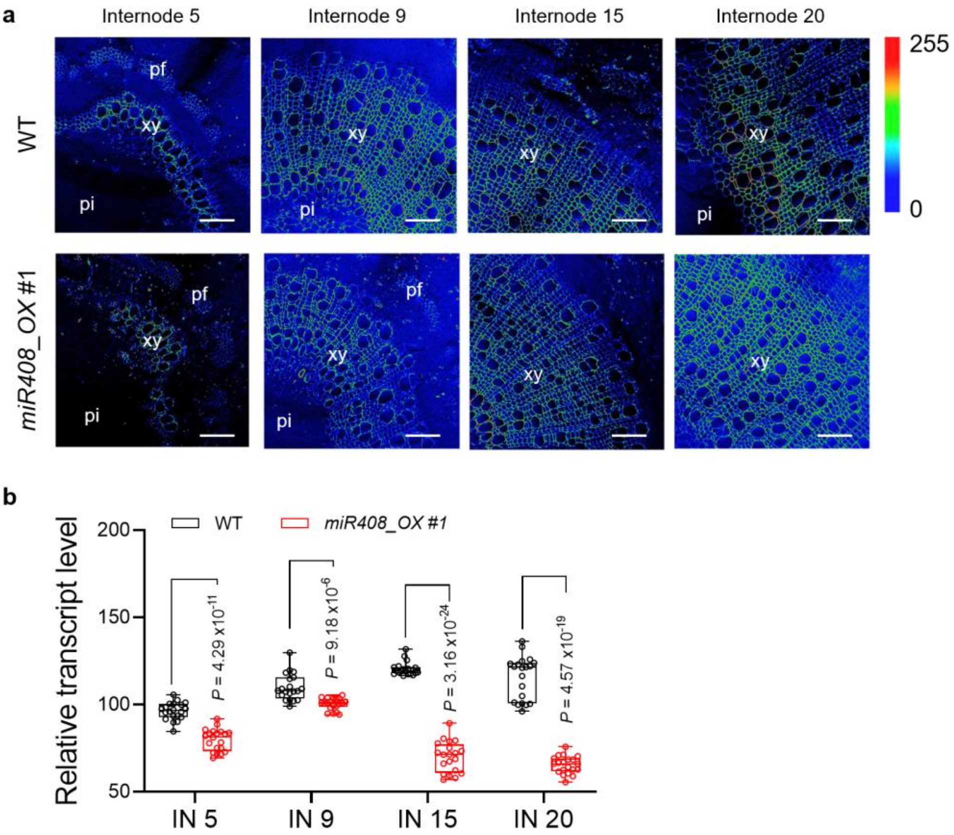

Supplementary Figure 10. SRS analysis of lignin in the secondary xylem cell wall. (**a**) Stimulated Raman mapping of WT and *miR408_OX* poplars. The *miR408_OX* plants exhibit a significant reduction in lignin content. xy, xylem; pi, pith. Scale bar, 100 μm. (**b**) SRS intensity is presented as pixel intensity and normalized to the average of all intensities, indicating lignin signal. The upper, lower and middle box lines represent the two quartiles and median of values in each group. n = 5, two-tailed Student's t-test, n represents 5 trees sampled from the *miR408_OX*, and 4 replicate cross sections were carried out for each transgenic line. Source data are provided as a Source Data file.

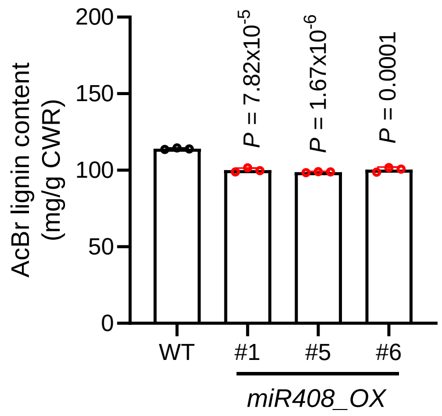

Supplementary Figure 11. AcBr lignin contents of stems of one-year-old *miR408_OX* and WT poplar. Values are means ± SD (*n* = 3, two-tailed Student's *t*-tests, n represents 3 trees sampled respectively from each transgenic line). Source data are provided as a Source Data file.

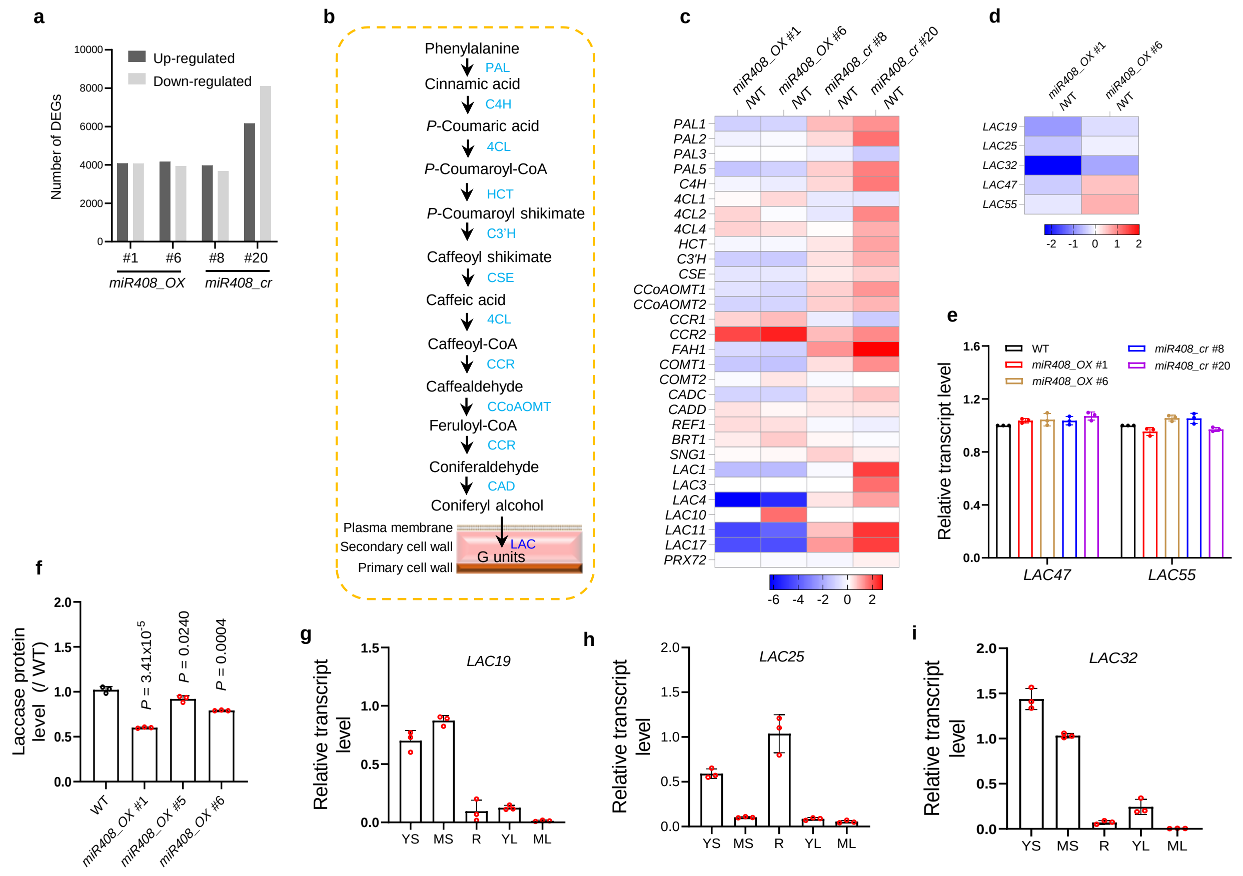

Supplementary Figure 12. Transcriptomic and qRT-PCR analyses of 3-month-old WT and *miR408_OX* and knockout plants.

(**a**) The number of up-regulated and down-regulated DEGs.

(**b-c**) Heatmap illustrating the differentially expressed genes in phenylpropanoid and secondary cell wall biosynthesis pathways. The genes encode hydroxycinnamoyl CoA: shikimate hydroxycinnamoyl transferase (*HCT*), 4-coumarate: CoA ligase (*4CL*), cinnamate 4-hydroxylase (*C4H*), L-phenylalanine ammonia-lyase (*PAL*), caffeic acid/5-hydroxyferulic acid 3-*O*-methyltransferase (*COMT*), ferulate/coniferaldehyde 5-hydroxylase (*F5H*), cinnamoyl CoA reducatse (*CCR*), caffeoyl CoA 3-*O*-methyltransferase (*CCoMT*), coumaroyl shikimate 3´-hydroxylase (*C3H*) and cinnamoyl CoA reductase (*CAD*).

(**d**) Heatmap illustrating the transcript levels of the five predicated target *LACs* from transcriptomic data.

(**e**) qRT-PCR showing the relative transcript levels of *LAC47* and *LAC55* in WT, *miR408_OX*, and knockout plants.

(**f**) Extractable laccase protein level in WT and *miR408_OX* plants. -

(**g-i**) Expression pattern of *LAC19*, *LAC25* and *LAC32* in different tissues in 6-month-old poplars. YS, young stem; MS, mature stem; R, root; YL, young leaves; ML, mature leaves. The numbers of individual trees sampled respectively from WT and each transgenic lines are as follows: e-i =3. Values are means ± SD. All *P*-values are from two-tailed Student’s *t*-tests. Source data are provided as a Source Data file.

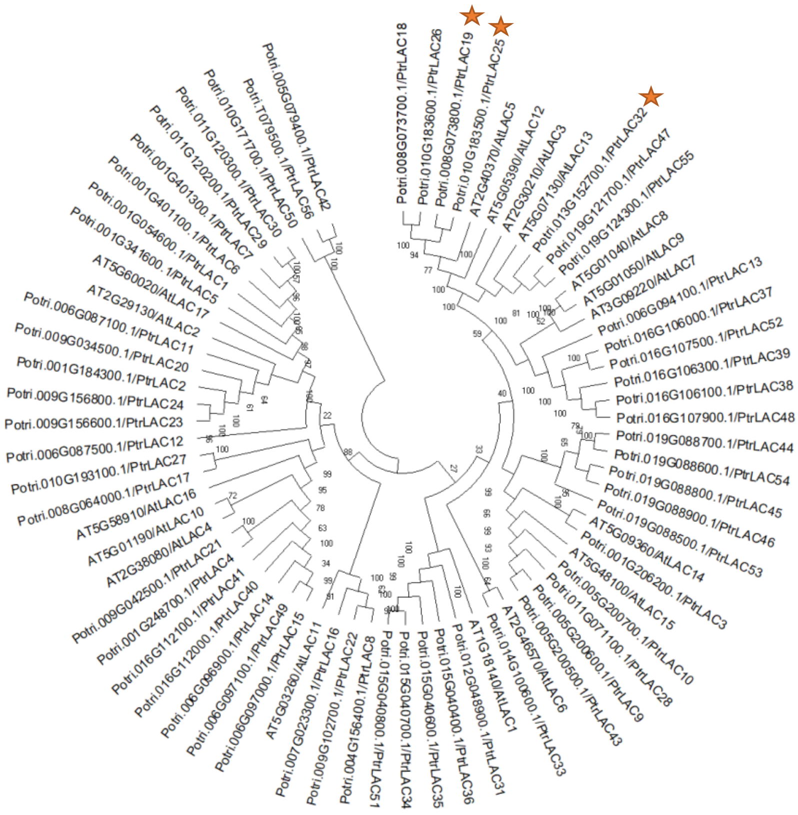

Supplementary Figure 13. Phylogenetic analysis of all the *LACs* between *P. trichocarpa* and *A. thaliana*. Yellow stars indicate the three target *LACs* of *miR408*. The aligned amino acid sequence used for phylogenetic analysis in Supplementary Figure 13 is presented in Supplementary Data 4.

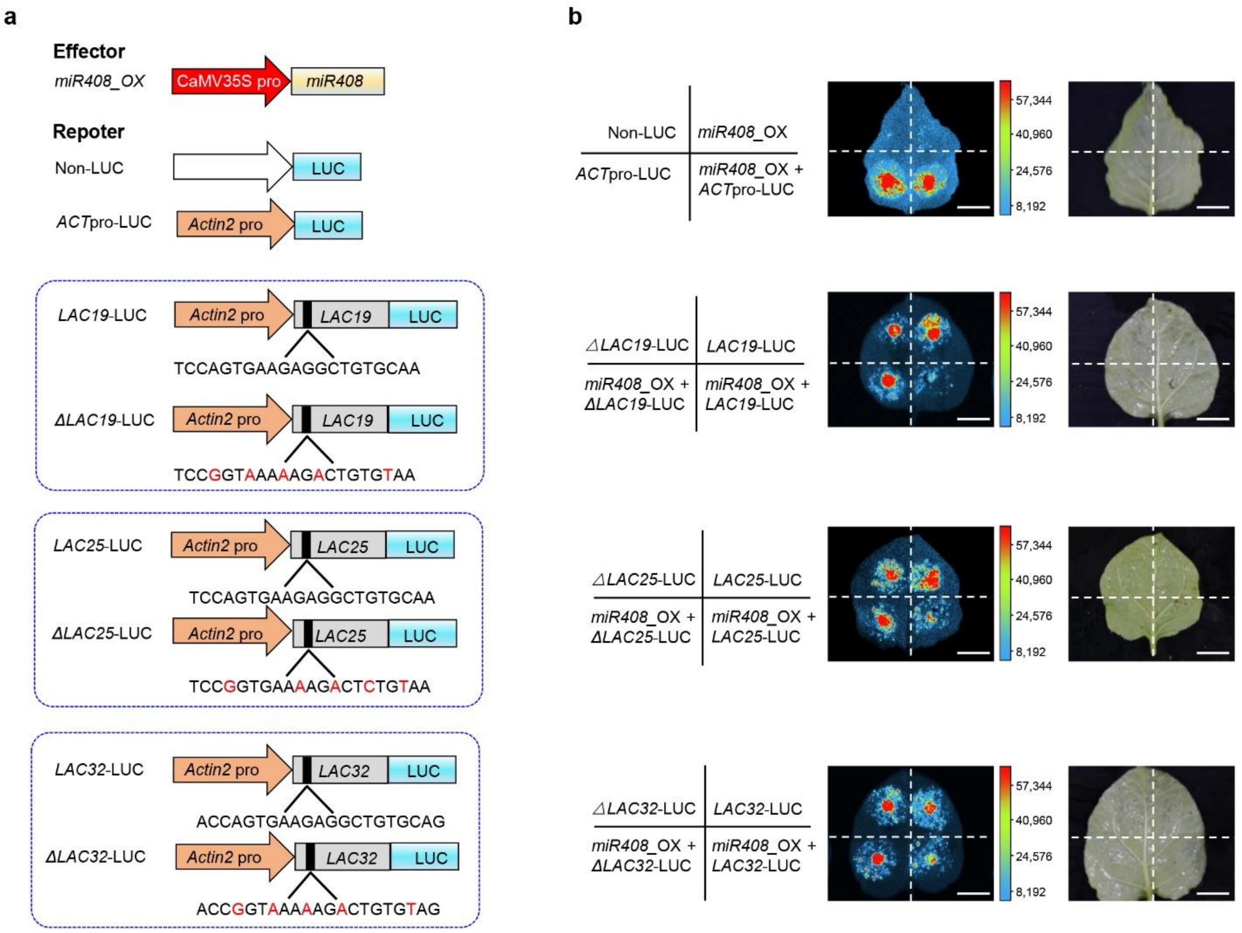

Supplementary Figure 14. Functional identification of targets of *miR408 in planta*.

(**a**) Vector constructs for luciferase assays. Five nucleotides were mutated at the predicted binding site of target *LACs* and *miR408*, in order to disrupt the *miR408* recognition site, while at the same time guaranteeing that the amino acid sequences were unchanged. The mutated CDSs of *LAC19*, *LAC25* and *LAC32* were named *ΔLAC19*, *ΔLAC25*, and *ΔLAC32*. The original predicted binding sites of *LAC19*, *LAC25* and *LAC32* were TCCAGTGAAGAGGCTGTGCAA, TCCAGTGAAGAGGCTGTGCAA and ACCAGTGAAGAGGCTGTGCAG, and the mutated binding sites of *ΔLAC19*, *ΔLAC25*, and *ΔLAC32* were TCCGGTAAAAAGACTGTGTAA, TCCGGTGAAAAGACTCTGTAA and ACCGGTAAAAAGACTGTGTAG, respectively. The complete CDSs of *LAC19*, *LAC25* and *LAC32* were fused to the LUC reporter. Control reporter constructs harbored the *ΔLAC19*, *ΔLAC25* and *ΔLAC32* mutants fused to LUC, LUC with an inactive promoter, and LUC constitutively expressed from the ACTIN promoter. Reporter constructs were co-infiltrated with CaMV35S-driven *miR408*.

(**b**) *miR408* suppressed the expression of *LAC19*, *LAC25* and *LAC32*. The constructs to express the indicated fusion proteins were transformed into *Nicotiana tabacum* leaves through *Agrobacterium* infiltration. Luciferase activity was visualized at 3 d after infiltration. Each leaf was divided into four parts. The right panels show the bright field photographs. Scale bar, 1 cm. Three samples each were analyzed with similar results.

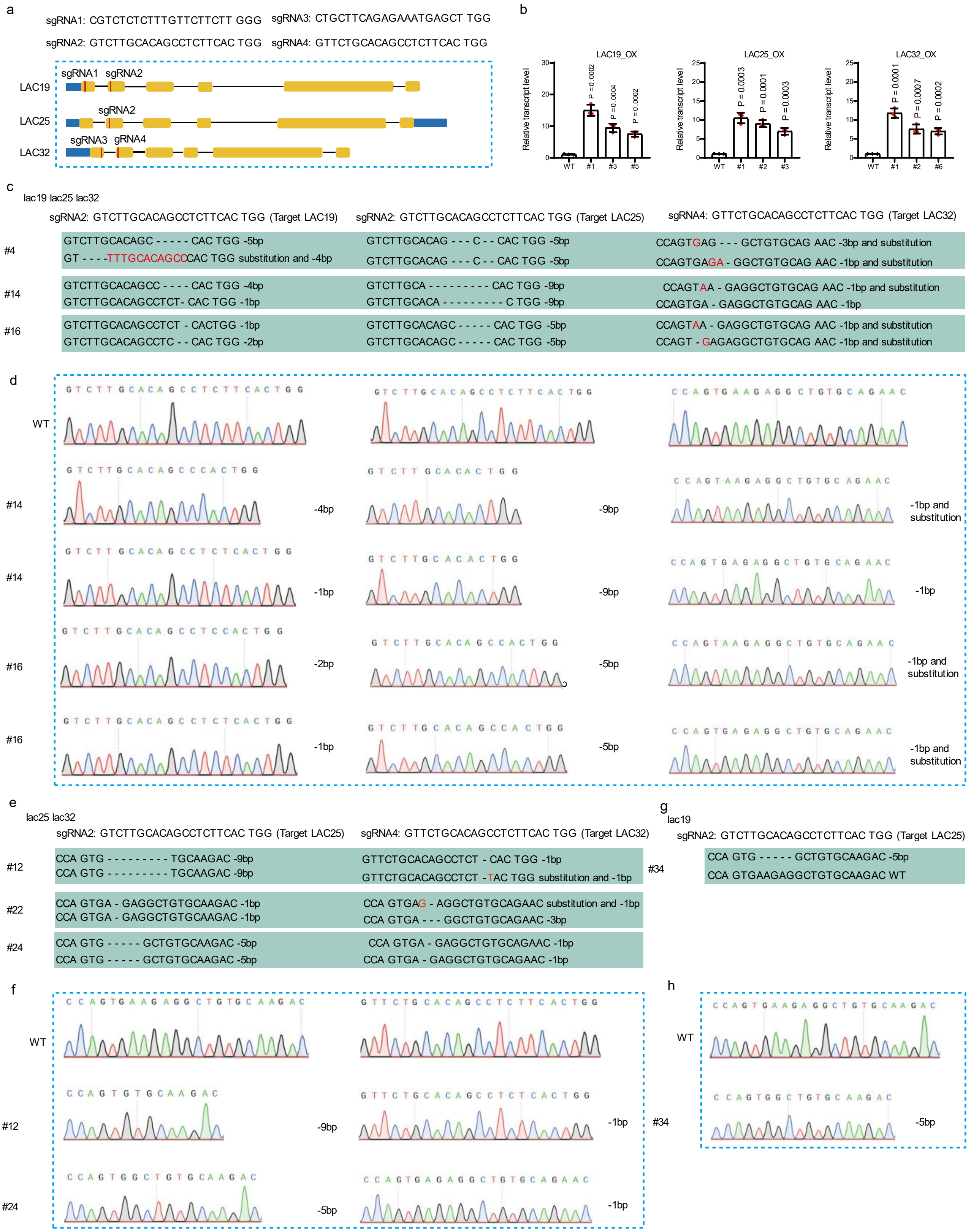

Supplementary Figure 15. Characterization of *LACs* knockout and overexpression poplar.
(a) The design of four sgRNAs. (**b**) The qRT-PCR analysis of the overexpression poplars. n represents 3 trees sampled respectively from each line, two-tailed Student's *t*-tests. Values are means ± SD. (**c-h**) Genotypes of *lac19 lac25 lac32* (**c-d**), *lac25 lac32* (**e-f**) *and lac19* (**g-h**) mutants generated by CRISPR–Cas9 DNA. Detailed sequence information is available in Supplementary Data 2. Source data are provided as a Source Data file.

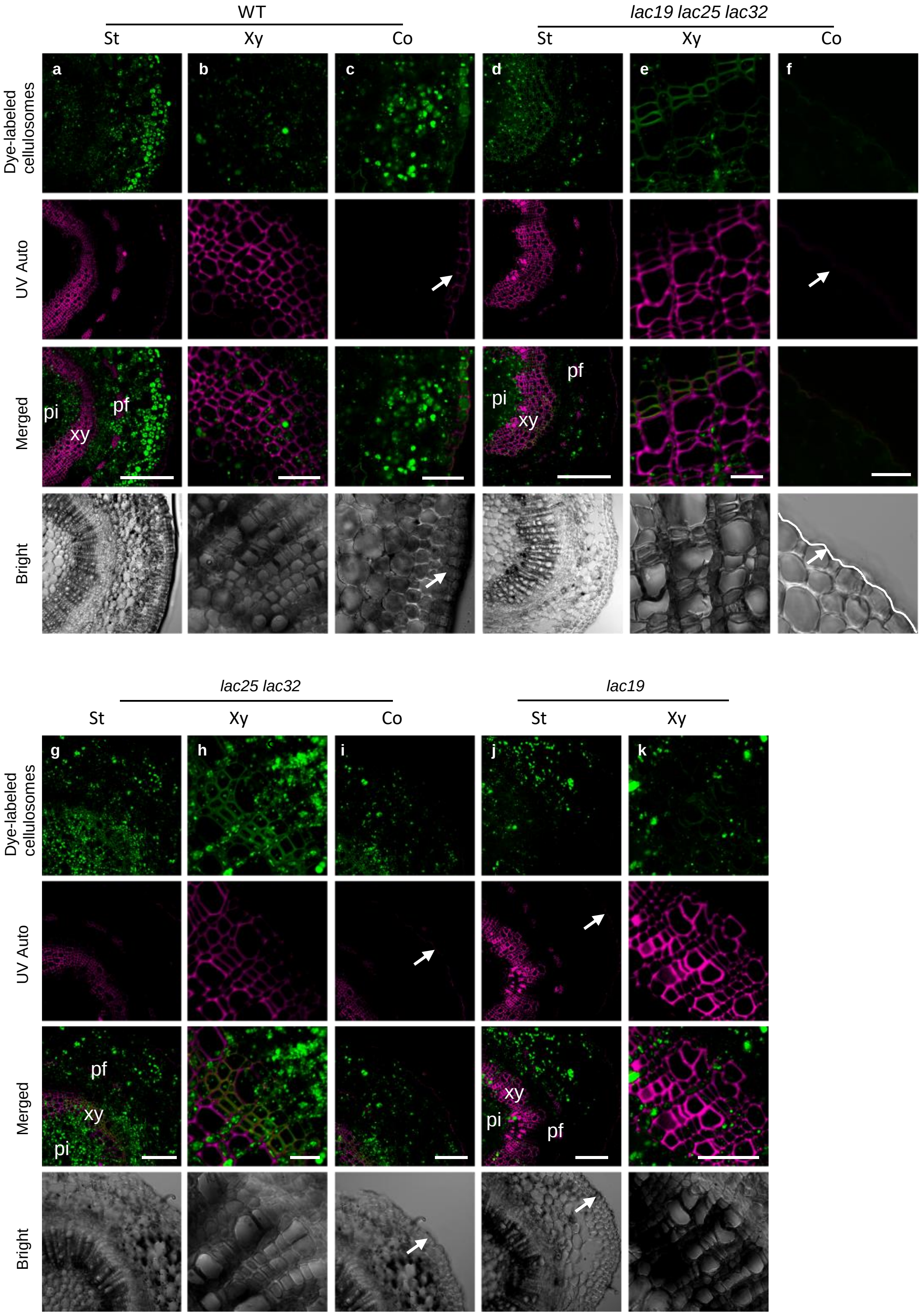

Supplementary Figure 16. Fluorescence microscopy of transverse sections of cell walls exposed to dye-bound cellulase in fresh stems of WT (a-c), *lac19 lac25 lac32* (d-f), *lac25 lac32* (g-i) *and lac19* (j-k). Cellulase bound to the cell walls appears as green fluorescence. The autofluorescence (red) under UV shows the presence of lignin. Autofluorescence (red) and overlay images highlight the negative correlation between probe binding and autofluorescence in the WT. After 30 min of incubation, the green signal can just be seen in intracellular material in the WT, but clearly in the cell wall of *lac19 lac25 lac32* and *lac25 lac32* stem. Pi, pith; xy, xylem; Co, cortex. Scale bar, 100 µm (**a**, **d, g, j**); 20 µm (**b-c**, **e-f, h-i, k**). Three samples each were analyzed with similar results for a-k. Arrows indicate the epidermis.

Supplementary Table 1. Summary of lignin subunits and linkages as revealed by 2D-HSQC NMR.

	%S	%G	S/G	%PB	%A	%B
WT	74.23	25.77	2.88	5.69	70.34	4.68
miR408_OX #1	66.44	33.56	1.98	9.19	65.45	4.73
miR408_OX #5	66.89	33.11	2.02	9.92	68.61	4.65
miR408_OX #6	68.85	31.15	2.21	10.87	67.08	4.88

[a] 2D-HSQC spectra of DEL samples isolated from 6-month-old WT and *miR408_OX* poplars. [b] S, syringyl; G, guaiacyl; PB, *p*-hydroxybenzoic; A, β–*O*–4 (β-aryl ether); B, β–β(phenylcoumaran).

[c] Results expressed per 100 Ar based on quantitative 2D-HSQC spectra.

[d] S/G ratio obtained by the equation: S/G ratio = 0.5I(S2,6)/I(G2).

Source data are provided as a Source Data file.

Supplementary Table 2. Cell wall lignin content of WT and *LAC_OX* poplars.

	**Lignin**		**Total lignin**
	**AIL**	**ASL**	
WT	23.13 ± 0.32	4.70 ± 0.20	27.83 ± 0.35
LAC19_OX	29.53 ± 0.35	3.77 ± 0.06	33.31 ± 0.29
LAC25_OX	27.00 ± 0.26	3.80 ± 0.05	30.80 ± 0.31
LAC32_OX	28.07 ± 0.55	3.83 ± 0.06	31.90 ± 0.61

Contents of acid insoluble lignin (AIL), acid soluble lignin (ASL), total lignin. Values are means ± SE (n = 3, n represents 3 trees sampled respectively from each transgenic line). Values are expressed as weight percent based on vacuum-dried extractive free wood weight (%, w/w).

Supplementary Table 3. Analysis of saccharification efficiency of one-year-old *laccase* mutant plants.

Digestion time	Sample name	Peak area	Glucose concentration (mg/mL)	Total glucose released (mg)	Saccharification efficiency
24h	WT	15665.33	0.17	17.04	21.69%
24h	*lac19 lac25 lac32* (#4)	33686.01	0.27	27.10	66.43%
24h	*lac19 lac25 lac32* (#14)	28873.03	0.24	24.41	45.25%
24h	*lac19 lac25 lac32* (#16)	35744.33	0.28	28.25	48.87%
24h	*lac25 lac32* (#12)	37009.02	0.28	28.96	32.73%
24h	*lac25 lac32* (#22)	24361.37	0.21	21.89	43.48%
24h	*lac25 lac32* (#24)	23234.10	0.21	21.27	44.77%
24h	*lac19* (#1)	21844.01	0.20	20.49	27.59%
24h	*lac19* (#2)	21919.67	0.21	20.53	26.91%
48h	WT	24939.07	0.22	22.22	28.28%
48h	*lac19 lac25 lac32* (#4)	43166.33	0.32	32.40	79.41%
48h	*lac19 lac25 lac32* (#14)	34840.01	0.28	27.74	51.43%
48h	*lac19 lac25 lac32* (#16)	42563.07	0.32	32.06	55.46%
48h	*lac25 lac32* (#12)	44735.02	0.33	33.27	37.61%
48h	*lac25 lac32* (#22)	35295.33	0.28	27.99	55.60%
48h	*lac25 lac32* (#24)	29899.11	0.25	24.97	52.60%
48h	*lac19* (#1)	28858.12	0.24	24.41	32.87%
48h	*lac19* (#2)	31822.33	0.26	26.06	34.16%
72h	WT	26214.67	0.23	22.93	29.19%
72h	*lac19 lac25 lac32* (#4)	43601.05	0.33	32.64	80.00%
72h	*lac19 lac25 lac32* (#14)	36420.33	0.29	28.63	53.06%
72h	*lac19 lac25 lac32* (#16)	53946.33	0.38	38.41	66.45%
72h	*lac25 lac32* (#12)	48086.67	0.35	35.14	39.73%
72h	*lac25 lac32* (#22)	36149.33	0.28	28.48	56.55%
72h	*lac25 lac32* (#24)	36510.05	0.29	28.68	60.37%
72h	*lac19* (#1)	30559.33	0.25	25.36	34.15%
72h	*lac19* (#2)	32338.67	0.26	26.35	34.53%
