# Supplementary material for: Manipulating microRNA miR408 enhances both biomass yield and saccharification efficiency in poplar
Source: Nat Commun. 2023 Jul 18;14:4285. doi: 10.1038/s41467-023-39930-3 (PMC10354043; doi:10.1038/s41467-023-39930-3)
Supplement: Supplementary file 3 — Description of Additional Supplementary Files [file 41467_2023_39930_MOESM3_ESM.pdf]

Description of Additional Supplementary Files

Supplementary Data 1. Predicated target genes of *miR408* in poplar.

Supplementary Data 2. Summary of *LAC* knockout sequences.

Supplementary Data 3. Oligonucleotide sequences for the primers and probes used in this study.

Supplementary Data 4. The aligned amino acid sequences used for phylogenetic analysis.
